# Associations of serum and tissue TIMP1 with host response and survival in colorectal cancer

**DOI:** 10.1038/s41598-025-85549-3

**Published:** 2025-01-09

**Authors:** Akseli Kehusmaa, Anne Tuomisto, Päivi Sirniö, Henna Karjalainen, Meeri Kastinen, Vilja V. Tapiainen, Ville K. Äijälä, Taina Tervahartiala, Timo Sorsa, Jukka Rintala, Sanna Meriläinen, Juha Saarnio, Tero Rautio, Markus J. Mäkinen, Juha P. Väyrynen

**Affiliations:** 1 Translational Medicine Research Unit, Medical Research Center Oulu, Oulu University Hospital, and University of Oulu, Aapistie 5A, 90220 Oulu, Finland; 2 Department of Oral and Maxillofacial Diseases, Institute of Dentistry, Helsinki University Central Hospital, Helsinki, Finland; 3 Department of Oral Diseases, Karolinska Institutet, Huddinge, Sweden

**Keywords:** Colorectal cancer, TIMP1, Prognosis, Immunohistochemistry, Serum biomarker, Colorectal cancer, Tumour biomarkers

## Abstract

Tissue inhibitor of matrix metalloproteinase 1 (TIMP1) is a multifaceted, cytokine-like bioactive molecule whose levels are elevated in a wide range of inflammatory diseases and are associated with prognosis. Additionally, TIMP1 may play a role in driving systemic inflammation. TIMP1 immunohistochemistry and TIMP1 serum concentrations were analyzed in a cohort of 776 colorectal cancer patients. TIMP1 histoscore by cell type (tumor cell, other) was quantified using digital image analysis. Serum TIMP1 levels were evaluated for correlations with tumor TIMP1 expression, and their associations with tumor characteristics, inflammation, and prognosis were investigated. High serum TIMP1 concentrations associated with shorter overall survival (multivariable HR 1.85, 95% CI 1.30–2.65). Serum TIMP1 levels positively correlated with markers of systemic inflammation and tumor necrosis percentage but not with TIMP1 expression in tumor tissue. High TIMP1 intensity in tumor stroma associated with longer cancer-specific and overall survival in univariable analysis but not in multivariable models. T cell densities in tumor tissue positively correlated with tumor stromal TIMP1 expression and negatively with tumor epithelial TIMP1 expression. Serum TIMP1 levels show promise as a prognostic marker for colorectal cancer and correlate with systemic inflammatory markers, but do not correlate with TIMP1 expression in tumor tissue.

**Supplementary Information:**

The online version contains supplementary material available at 10.1038/s41598-025-85549-3.

## Introduction

Tissue inhibitor of metalloproteinases 1 (TIMP1) is a multifactorial molecule with both protease inhibitor and cytokine-like activities. As a member of the TIMP family, it inhibits matrix metalloproteinases (MMPs), enzymes that degrade extracellular matrix components such as collagen and elastin^1^. Emerging evidence highlights the cytokine functions of TIMP1, mediated through interactions with at least 18 partners, including membrane receptors^2^. Beyond its protease-inhibitory role, TIMP1 regulates angiogenesis, cell cycle, and differentiation, while also exhibiting anti-apoptotic activities^3^. Elevated circulating or tissue TIMP1 levels have been observed in numerous inflammatory and cancer diseases, and they have been associated with poor prognosis^4–8^.

Colorectal cancer (CRC) is the third most common cancer disease globally, with 1.9 million new cases in 2020^9^. Novel serum and tissue biomarkers are needed for early detection and accurate prognostication of the disease, and high serum TIMP1 concentrations have been reported to predict worse prognosis^2,6^. In addition, high TIMP1 expression on tumor cells has also been associated with worse prognosis, as well as with changes in immune infiltration^7,8,10^. In cancer tissue, TIMP1 is produced by tumor cells, myofibroblasts and various immune cells. Its multifunctional roles include promoting tumor metastasis and cell proliferation, exhibiting anti-apoptotic effects, and modulating angiogenesis^5,11,12^. Additionally, TIMP1 can influence immune cell activities, potentially shaping tumor-associated immune reactions^13^. Larger-scale studies are required to further elucidate the biological roles of TIMP1 and confirm its utility as a prognostic indicator.

Few studies have comprehensively assessed TIMP1 expression in CRC tissue, especially its distribution in tumor epithelium and stroma^8^. Furthermore, no prior studies have examined the relationship between tumor TIMP1 expression and circulating TIMP1 levels. In this study, we analyzed TIMP1 expression in tumor epithelial and stromal cells, as well as TIMP1 serum levels, in a cohort of 776 CRC patients (606 with preoperative serum TIMP1 data and 757 with tissue TIMP1 data). We explored the correlation between serum and tumor TIMP1 levels and investigated their associations with tumor characteristics, inflammation, and prognosis. TIMP1 expression in tumor epithelial and stromal cells was analyzed with immunohistochemistry (IHC) combined with supervised machine learning based image analysis, facilitating consistent analysis across a large number of cases.

## Methods

### Study population

The study included all CRC patients who underwent tumor resection in Oulu University Hospital during 2006–2020 and provided informed consent for participation, including sample collection (*N* = 1011). A flowchart representing the patient selection process is shown in Fig. S1. The study cohort is being prospectively collected and has previously been described for the years 2006–2014 and now extends to 2020^14,15^. Patients who had received preoperative chemotherapy or radiotherapy (*N* = 235) were excluded from further analyses, after which data of 776 patients could be utilized (of which 757 with tissue TIMP1 data and 606 with serum TIMP1 data). Both serum and tissue TIMP1 data were available for 591 patients. For survival analyses, the patients who died within 30 days of surgery (*N* = 5 for tissue data, *N* = 4 for serum data) were additionally excluded to account for probable surgery-related deaths.

Patients had follow-up data until December 31, 2021, with a median follow-up time of 5.6 years (IQR 3.7–9.3) for censored cases. Up to 10 years of follow-up data were used, as most CRC-related deaths occur within this timeframe^16^. Follow-up data were collected from clinical records and Statistics Finland. The primary endpoints were cancer-specific survival (CSS), defined as the time from surgery to death from the same cancer, and overall survival (OS), defined as the time from surgery to death from any cause. Clinical data, including preoperative American Association of Anesthesiologists (ASA) classification (estimate of overall disease burden and fitness for anesthesia) and comorbidities (diabetes, coronary artery disease, asthma, and COPD), were collected as a part of standard care, and recovered from medical records. All patients provided informed written consent to participate in the study. The REporting recommendations for tumor MARKer prognostic studies (REMARK) were taken into account in the study design^17^.

### Histopathological analysis

Heamatoxylin & eosin (H&E)-stained tumor sections were used for histopathological evaluation. TNM stage was determined using the UICC/AJCC (Union for International Cancer Control/The American Joint Committee on Cancer) criteria and tumor grade using the World Health Organization criteria. Lymphovascular invasion and the percentage of tumor necrosis were visually estimated^18,19^.

### Tissue microarray construction

Tissue microarrays (TMAs) were constructed from formalin-fixed paraffin-embedded (FFPE) tissue samples with either Galileo TMA CK4500 (Integrated Systems Engineering, Milan, Italy) or TMA Master II (3DHistech Ltd., Budapest, Hungary)^20^. Four 1.0-mm diameter cores were taken from each tumor: two from the center of the tumor (CT) and two from the invasive margin (IM).

### Immunohistochemistry and image analysis

TMA blocks were sectioned at 3.5 μm and immunohistochemically stained for TIMP1 using an anti-TIMP1 antibody [EPR18352] (Abcam), diluted 1:4000 and incubated for 30 min at room temperature. Staining was performed on a BOND RX instrument (Leica Biosystems) with Bond Polymer Refine Detection Kit (Leica Biosystems, DS9800). Antigen retrieval was performed for 30 min at 100 °C using BOND Epitope Retrieval Solution 2 (Leica Biosystems, AR9640).

**Fig. 1:**
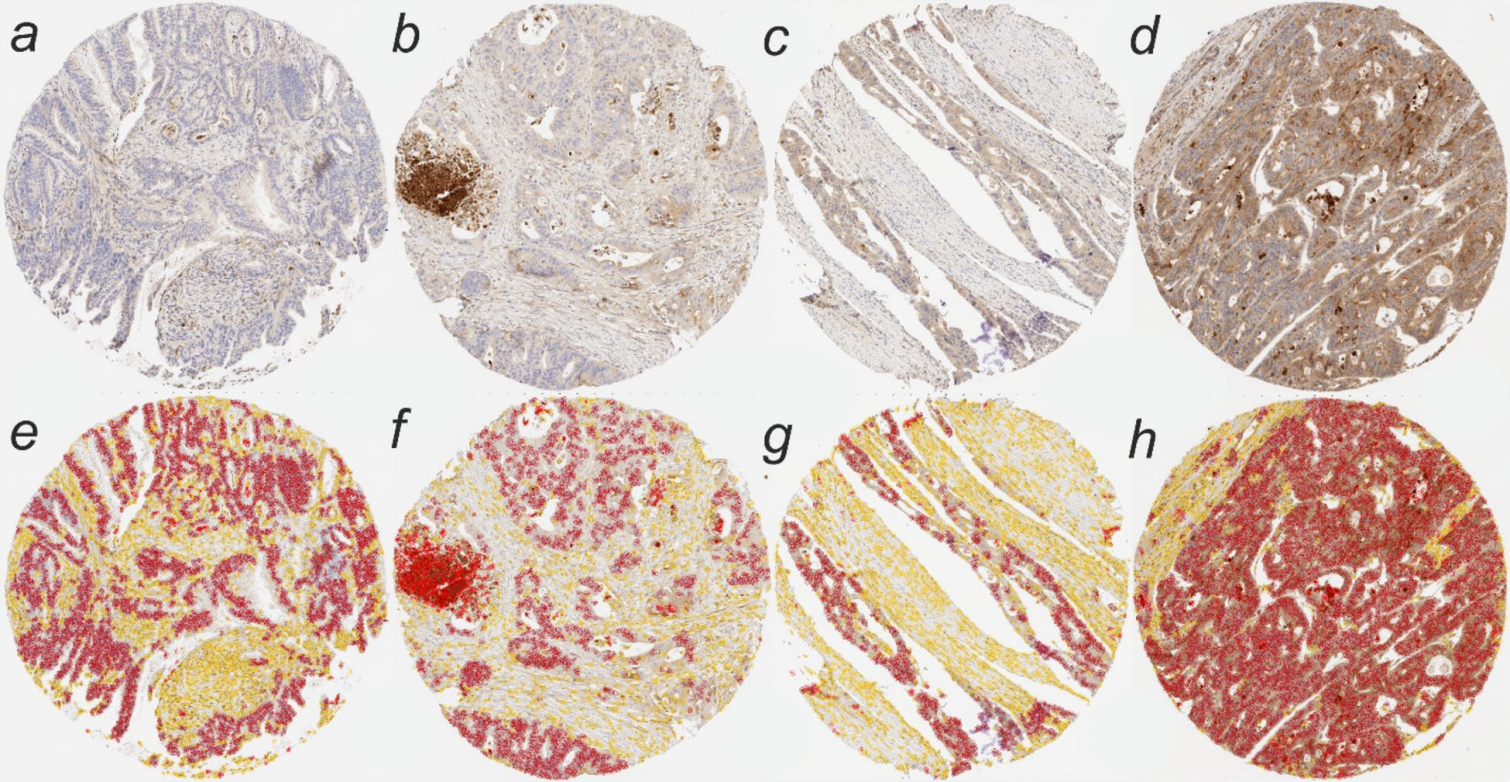
Examples of TIMP1 immunohistochemistry images and image analysis result images. (**a–d**) Examples of four tissue microarray (TMA) cores with increasing tumor TIMP1 staining intensity. (**e–h**) Cell detection and classification of the four TMA cores using QuPath. Dark red denotes cancer cells, yellow other cells, and bright red cells in necrotic areas (as e.g. in **f**), which were ignored from the analyses.

Digital image analysis was conducted using QuPath (version 0.4.3), an open source bioimage analysis software^21^. The previously validated machine-learning algorithms of QuPath were used to detect individual cells, calculate additional features (Haralick’s features and smoothed features) and train an *object classifier* to recognize tumor cells, stromal cells (including immune cells), and cells in necrotic regions (excluded from analysis) by annotating representative areas^22^. The cores with insufficient material were excluded. QuPath was used to measure the chromogen (3,3’-Diaminobenzide, DAB) intensity along with other features for each cell. The process of the automatic classification was supervised. Examples of TMA cores with TIMP1 staining and respective cell classifications can be seen in Fig. 1.

We processed the cell data using RStudio (version 2023.06.02) and R statistical programming (version 4.3.1, R core Team). For each cell, cytoplasmic DAB intensity was classified into four categories; DAB < 0.1 intensity units indicated negative (0), DAB 0.1–0.15 indicated weakly positive (1), DAB 0.15–0.3 indicated moderately positive (2) and DAB > 0.3 indicated strongly positive (3). The intensity values were used to calculate TIMP1 histoscores for each core, separately for cancer and stromal cells, using the formula: TIMP1 histoscore = 100 × (3 × percentage of strong positive cells + 2 × percentage of moderate positive cells + 1 × percentage of weak positive cells)^23^. For most downstream analyses, a single histoscore was used for each tumor, calculated as the average of histoscores from all individual cores. TIMP1 histoscores specific to the CT and IM were calculated as the averages of the respective cores and could be defined for 754 and 745 patients, respectively.

To assess the accuracy of the image analysis algorithm, we manually annotated cells on 30 TMA cores from consecutive tumors alongside performing automatic classification. Among cells manually annotated as tumor cells, the machine-learning algorithms correctly classified 86% correctly as tumor. Similarly, 88% of stromal cells were accurately recognized by the algorithm. To further evaluate the reliability of the results, histoscores were calculated for these 30 validation TMA cores using three methods: (1) automated classification by QuPath, (2) manual annotation-based evaluation using QuPath, and (3) fully manual visual assessment. The histoscores generated by the automated classifier showed strong correlations with those derived by manual annotations of tumor regions (*r* = 0.99 for epithelial cell histoscore and *r* = 0.93 for stromal cell histoscore). Additionally, the automated histoscores demonstrated a high correlation with visually evaluated histoscores for epithelial cells (*r* = 0.79) and a moderate correlation for stromal cells (*r* = 0.54).

TMA cores were stained for CD3 and CD8, and T cell densities were calculated, as described before^20^. *BRAF* and mismatch repair (MMR) status of the tumors were defined from the TMA cores by IHC^20^.

### Blood and serum analysis

Preoperative venous blood samples were collected and centrifuged for serum extraction. Samples were stored at − 70 °C. Blood neutrophil and lymphocyte counts, and C-reactive protein (CRP) and albumin concentrations were measured in the laboratory of Oulu University Hospital. The analysis was successful for 730, 730, 722, and 766 cases, respectively^12^. The modified Glasgow prognostic Score (mGPS) was determined based on the serum CRP and albumin levels; mGPS 0 indicated no changes in CRP (≤ 10 mg/L), mGPS 1 indicated an increase of CRP (> 10 mg/L) without changes in albumin levels (≥ 35 g/L), and mGPS 2 indicated both an increase in CRP (> 10 mg/L) and decrease in albumin (< 35 g/L) levels. TIMP1 was analyzed using TIMP1 enzyme-linked immunosorbent assay (ELISA) (R&D Systems, Minneapolis, MN) from 606 patients between 2010 and 2020^24^. Serum IL6 analyses were performed by Olink Target 96 Immuno-Oncology panel^18^, and the analysis was successful for 603 cases between 2010 and 2020.

### Statistical analyses

Statistical analyses were performed using IBM SPSS Statistics for Windows (IBM Corp. version 29.0). Findings with two-sided *p* < 0.05 were considered statistically significant. The statistical significance of the associations between categorical and continuous variables were analyzed using Mann-Whitney U test for variables with two categories, and Kruskal-Wallis test for variables with three or more categories. Spearman correlation coefficients were used to assess the correlation between the manually derived estimates of TIMP1 expression and the results of the supervised machine-learning algorithm for the 30 validation TMA cores. Pearson correlation coefficients were used to assess the correlation between serum TIMP1 values, TIMP1 histoscores (cancer cells and stromal cells), T cell densities, tumor necrosis percentage, blood cell counts, and serum inflammatory markers. The normality of the data was assessed visually using histograms, and variables with positive skewness were normalized through logarithmic transformation. Linear regression models were used to adjust the correlation coefficients for the following covariates: age (< 65, ≥ 65), sex (male, female), tumor localization (colon, rectum), T class (T1-T2, T3-T4), N class (N0, N1-N2), M class (M0, M1), *BRAF* status (wild-type, mutant), MMR status (proficient, deficient), and tumor grade (low, high). The assumptions of linear regression model (linearity, normality, and homoscedasticity) were examined using a normal probability plot of residuals and a scatterplot of residuals versus predicted values.

Receiver operating characteristics (ROC) analysis was performed for TIMP1 serum values and TIMP1 histoscores. The area under the curve (AUC) from the ROC analysis was used to assess the capacity of the variables to predict 10-year CSS and OS. An optimal cut-off value was determined based on the longest distance from the diagonal reference line^23^. Kaplan-Meier curves were drawn to visualize survival in groups defined by the chosen cut-off values, and statistical significance was tested by using the log rank test. We used Cox proportional hazard regression models to estimate the hazard ratios (HRs) for TIMP1 serum values and TIMP1 histoscores in relation to CSS and OS. Multivariable Cox proportional hazards regression models were adjusted for sex, age (< 65, 65–75, > 75), year of operation (2006–2010, 2011–2015, 2016–2020), tumor location (proximal colon, distal colon, rectum), disease stage (I–II, III, IV), tumor grade (low-grade, high-grade), lymphovascular invasion (negative, positive), MMR status (proficient, deficient), and *BRAF* status (wild-type, mutant). The proportionality of hazards was examined by plotting the Shoenfeld residuals, which supported the proportionality during most of the follow-up period up to 10 years.

## Results

### Serum and tissue TIMP1 in relation to clinicopathological features

Serum TIMP1 concentrations were successfully measured for 606 patients, and TIMP1 immunohistochemistry was successful for 757 patients (Table 1). In immunohistochemical analysis, anti-TIMP1 antibody labeled tumor epithelial cells with varying intensities, necrosis strongly, and stroma (including immune cells) more diffusely with generally weaker intensity (Fig. 1). We separately measured TIMP1 expression in tumor epithelial cells and stromal cells using the histoscore method, with tumor epithelial and stromal histoscores showing positive correlation (*r* = 0.76, *p* < 0.001).

**Table 1:** Demographic and clinical characteristics of colorectal cancer cases according to TIMP1 serum levels and histoscore values in tumor cells and other cells in tumor tissue.

Characteristic	TIMP1 histoscore in tumors					TIMP1 serum		
	Total *N*	TIMP1 tumor cells Median (IQR)	*P*	TIMP1 stromal cells Median (IQR)	*P*	Total *N*	TIMP1 serum ng/ml Median (IQR)	*P*
All cases	757 (100%)	111 (66–156)		81 (53–110)		606 (100%)	317 (268–376)	
Sex			0.62		0.69			0.15
Male	401 (53%)	106 (67–155)		82 (54–106)		330 (54%)	312 (262–376)	
Female	356 (47%)	112 (66–158)		80 (52–114)		276 (46%)	322 (276–377)	
Age (years)			0.63		0.93			< 0.001
< 65	228 (30%)	111 (66–159)		81 (52–114)		175 (29%)	292 (252–360)	
65–75	279 (37%)	111 (71–158)		81 (55–111)		226 (37%)	320 (275–380)	
> 75	250 (33%)	109 (60–153)		81 (53–107)		205 (34%)	333 (273–397)	
ASA classification			0.38		0.499			< 0.001
1	39 (5%)	117 (80–166)		77 (52–109)		32 (5%)	282 (241–330)	
2	287 (38%)	111 (71–156)		84 (57–119)		242 (40%)	313 (263–369)	
3	312 (41%)	110 (72–157)		83 (54–108)		252 (41%)	316 (269–375)	
4	61 (8%)	130 (75–172)		84 (62–110)		51 (9%)	372 (304–444)	
Missing data	58 (8%)					29 (5%)		
Tumor location			< 0.001		< 0.001			0.032
Proximal colon	315 (42%)	125 (74–170)		91 (63–126)		252 (42%)	330 (276–380)	
Distal colon	204 (27%)	98 (54–145)		70 (48–98)		165 (27%)	317 (272–382)	
Rectum	238 (31%)	106 (72–147)		76 (50–101)		189 (31%)	302 (259–358)	
AJCC disease stage			0.49		0.047			0.12
I	174 (23%)	104 (59–152)		84 (57–111)		156 (26%)	310 (261–372)	
II	251 (33%)	114 (72–163)		84 (56–111)		188 (31%)	317 (272–376)	
III	248 (33%)	108 (66–159)		79 (53–113)		204 (34%)	313 (270–366)	
IV	84 (11%)	113 (58–154)		67 (45–100)		58 (9%)	350 (273–434)	
Tumor grade			0.027		0.011			0.054
Low-grade	651 (86%)	106 (66–152)		80 (53–107)		518 (85%)	313 (265–376)	
High-grade	106 (14%)	126 (71–171)		86 (57–130)		88 (15%)	334 (284–396)	
Lymphovascular invasion			0.28		0.024			0.83
No	413 (55%)	112 (68–157)		84 (57–113)		338 (56%)	313 (270–377)	
Yes	344 (45%)	108 (65–154)		77 (49–107)		268 (44%)	320 (259–375)	
MMR status			< 0.001		< 0.001			< 0.001
MMR proficient	639 (84%)	105 (61–149)		77 (51–104)		509 (84%)	311 (263–372)	
MMR deficient	118 (16%)	152 (94–185)		109 (78–138)		97 (16%)	346 (294–401)	
*BRAF* status			< 0.001		< 0.001			0.0064
Wild-type	651 (86%)	104 (60–146)		77 (51–104)		513 (85%)	312 (266–372)	
Mutant	106 (14%)	158 (124–207)		115 (86–136)		87 (14%)	342 (293–400)	
Missing data						6 (1%)		
mGPS			< 0.001		0.009			< 0.001
0	554 (73%)	106 (60–151)		79 (52–107)		469 (77%)	303 (256–354)	
1	96 (13%)	106 (71–182)		78 (50–109)		77 (13%)	377 (302–427)	
2	53 (7%)	146 (105–173)		95 (67–126)		51 (9%)	387 (333–474)	
Missing data	54 (7%)					9 (1%)		

*ASA* American Society of Aneshesiologists, *AJCC* American Joint Committee on Cancer, *mGPS* modified Glasgow prognostic score, *MMR* mismatch repair.

The association of TIMP1 serum levels and tissue expression with key clinicopathological features was assessed (Table 1). The median age of patients was 71 years (range: 30–92). High TIMP1 serum levels associated with MMR deficient status, high ASA classification, older age, elevated mGPS (all *p* < 0.001), proximal tumor location (*p* = 0.032), and *BRAF* mutation (*p* = 0.006). High TIMP1 histoscore in both tumor epithelial and stromal cells strongly associated with proximal tumor location, MMR deficient status, and *BRAF* mutation (all *p* < 0.001). Additionally, high TIMP1 histoscore in tumor cells associated with high mGPS (*p* < 0.001), while high TIMP1 histoscore in stromal cells associated with high mGPS (*p* = 0.009) and lower stage (*p* = 0.047). No associations were observed between TIMP1 levels (serum or tissue) and the analyzed comorbidities (Table S1).

### TIMP1 immunohistochemistry in relation to inflammatory tumor microenvironment

To explore the potential role of TIMP1 in tumor-host interactions, we analyzed the correlations of TIMP1 expression in tumor tissue with T cell densities, tumor necrosis percentage, systemic inflammation markers, and blood cell counts (Table 2). TIMP1 histoscore in tumor and stromal cells showed distinct correlations with T cell densities in a consistent pattern across the whole tumor, CT, and IM levels (Table S2). TIMP1 histoscore in tumor cells negatively correlated with T cells located in the CT. For example, for stromal CD3^+^ cell density, the multivariable beta coefficient was − 0.16 (*p* < 0.001). Conversely, TIMP1 histoscore in stromal cells did not significantly correlate with T cell densities in the CT, but positively correlated with T cell densities in the IM and overall tumor regions. For example, overall CD8^+^ cell density had a multivariable beta 0.13 (*p* < 0.001).

**Table 2:** Correlations between TIMP1 histoscore in tumors (tumor cells and stromal cells), TIMP1 serum levels, histological features, and systemic inflammation markers.

Variable	TIMP1 histoscore in tumors									TIMP1 serum				
	*N*	TIMP1 tumor cells				TIMP1 stromal cells				*N*				
		Unadjusted		Adjusted		Unadjusted		Adjusted			Unadjusted		Adjusted	
		Pearson r	p value	Beta	p value	Pearson r	p value	Beta	p value		Pearson r	p value	Beta	p value
TIMP1 histoscore tumor cells	757	1	–	–	–	0.76	< 0.001	0.74	< 0.001	591	0.016	0.69	− 0.021	0.62
TIMP1 histoscore stromal cells	757	0.76	< 0.001	0.74	< 0.001	1	–	–	–	591	0.016	0.69	− 0.014	0.75
CD3 + T cell overall density	756	0.032	0.39	− 0.010	0.79	0.14	< 0.001	0.085	0.026	594	− 0.061	0.14	− 0.051	0.25
CD3 + T cell density (CT, epithelial)	755	− 0.070	0.054	− 0.14	< 0.001	0.021	0.56	− 0.061	0.11	594	− 0.119	0.004	− 0.13	0.004
CD3 + T cell density (CT, stromal)	755	− 0.14	< 0.001	− 0.16	< 0.001	− 0.054	0.14	− 0.082	0.029	594	− 0.080	0.051	− 0.051	0.24
CD3 + T cell density (IM, epithelial)	749	0.014	0.70	− 0.023	0.55	0.12	0.001	0.066	0.079	589	− 0.089	0.031	− 0.090	0.40
CD3 + T cell density (IM, stromal)	749	0.00	0.99	− 0.022	0.56	0.14	< 0.001	0.11	0.005	589	− 0.056	0.17	− 0.041	0.34
CD8 + T cell overall density	756	0.060	0.10	− 0.011	0.78	0.20	< 0.001	0.13	< 0.001	594	− 0.015	0.71	− 0.031	0.48
CD8 + T cell density (CT, epithelial)	755	− 0.030	0.40	− 0.10	0.008	0.079	0.029	− 0.009	0.81	594	− 0.046	0.26	− 0.054	0.23
CD8 + T cell density (CT, stromal)	755	− 0.044	0.23	− 0.10	0.006	0.083	0.023	0.012	0.76	594	− 0.008	0.86	− 0.019	0.66
CD8 + T cell density (IM, epithelial)	751	0.010	0.78	− 0.052	0.18	0.10	< 0.001	0.021	0.58	590	− 0.041	0.32	− 0.047	0.29
CD8 + T cell density (IM, stromal)	751	0.033	0.36	− 0.013	0.73	0.18	< 0.001	0.13	< 0.001	590	− 0.011	0.80	− 0.012	0.79
Tumor necrosis percentage	757	− 0.16	< 0.001	− 0.14	< 0.001	− 0.069	0.057	− 0.016	0.68	606	0.10	0.012	0.091	0.034
Blood neutrophil count	712	0.014	0.71	− 0.016	0.67	− 0.051	0.18	− 0.082	0.026	595	0.25	< 0.001	0.22	< 0.001
Blood lymphocyte count	712	0.002	0.95	− 0.008	0.82	− 0.079	0.035	− 0.085	0.018	595	− 0.059	0.15	− 0.052	0.20
NLR	712	0.014	0.72	− 0.002	0.97	0.023	0.55	0.005	0.89	595	0.23	< 0.001	0.20	< 0.001
Serum CRP	704	0.087	0.021	0.049	0.20	0.070	0.065	0.043	0.26	598	0.43	< 0.001	0.41	< 0.001
Serum albumin	747	− 0.15	< 0.001	− 0.12	< 0.001	− 0.19	< 0.001	− 0.17	< 0.001	597	− 0.23	< 0.001	− 0.19	< 0.001
Serum IL6	588	0.041	0.32	0.006	0.88	0.031	0.46	0.005	0.91	603	0.44	< 0.001	0.41	< 0.001

The adjusted beta coefficients and P values were calculated with linear regression models that included age (< 65, ≥ 65), sex (male, female), tumor localization (colon, rectum), T class (T1–T2, T3–T4), N class (N0, N1–N2), M class (M0, M1), *BRAF* status (wild-type, mutant), MMR status (proficient, deficient), and tumor grade (low, high). Continuous variables with positive skewness were logarithmically transformed.

*CT* tumor center, *CRP* C-reactive protein, *IL* interleukin, *IM* invasive margin, *NLR* neutrophil-to-lymphocyte ratio.

Tumor necrosis percentage negatively correlated with TIMP1 histoscore in tumor cells (beta=-0.14, *p* < 0.001). Among systemic inflammatory markers and blood cell counts, serum albumin levels inversely correlated with TIMP1 histoscores in both tumor and stromal cells (*p* < 0.001 for both). TIMP1 histoscore in neither tumor nor stromal cells showed statistically significant correlation with serum TIMP1 levels.

### TIMP1 serum values in relation to systemic inflammation

We also explored the correlations between serum TIMP1 concentrations and systemic inflammatory markers (Table 2). Serum TIMP1 levels positively correlated with blood neutrophil count (beta = 0.22, *p* < 0.001), but not with lymphocyte count. Positive correlations were also found between serum TIMP1 levels and markers of systemic inflammation, including neutrophil-to-lymphocyte ratio (NLR), CRP and IL6 (all *p* < 0.001). The strongest correlations were observed with CRP and IL6 (beta = 0.41 for both). Serum TIMP1 levels also showed a weak positive correlation with tumor necrosis percentage (beta = 0.091, *p* = 0.034).

### Survival analyses

**Fig. 2:**
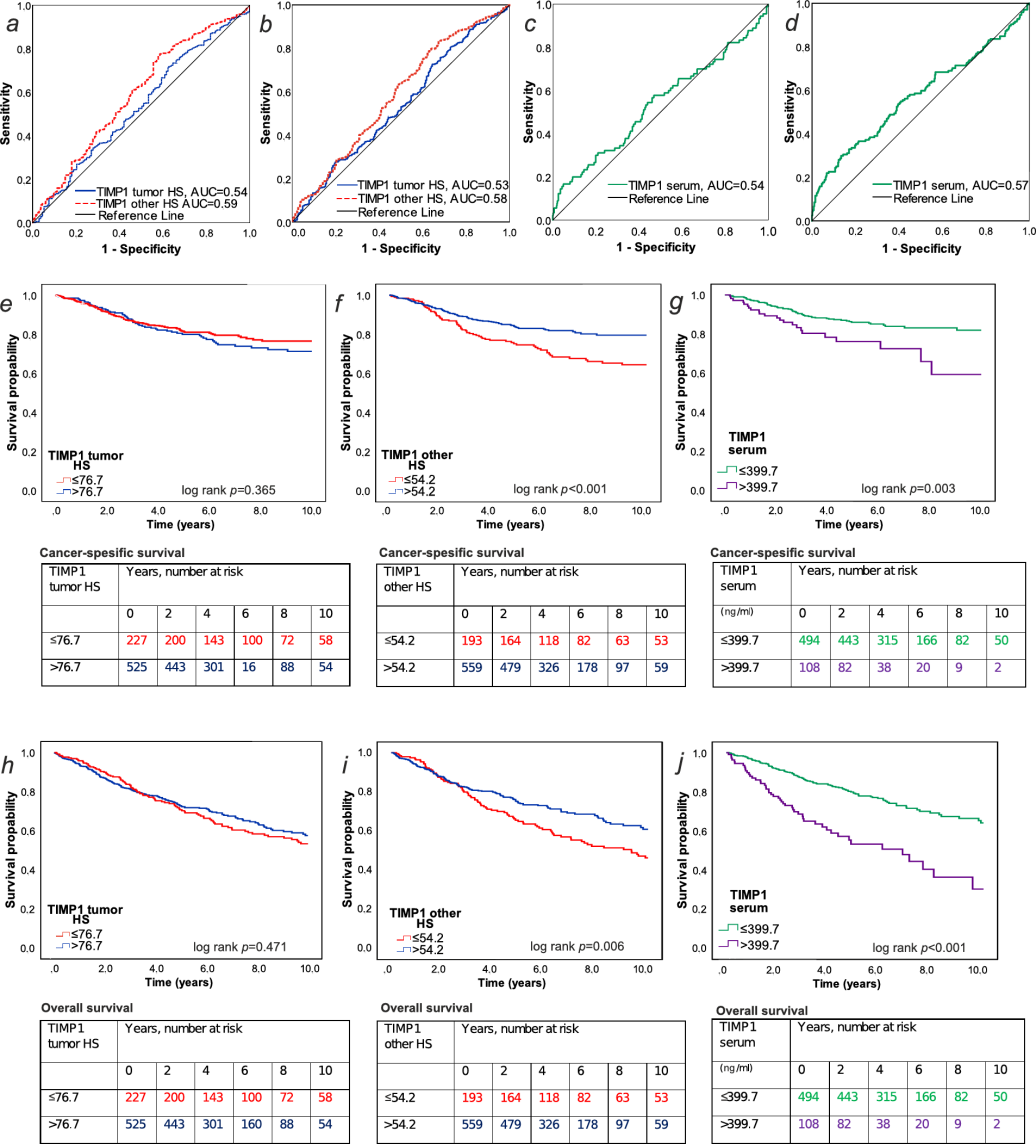
Receiver operating characteristics (ROC) and Kaplan-Meier survival analyses. (**a**,**b**) ROC curves for TIMP1 histoscore in tumor and stromal cells for (**a**) cancer-specific survival (CSS) and (**b**) overall survival (OS). (**c**,**d**) ROC curves for TIMP1 serum concentrations for (**c**) CSS and (**d**) OS. (**e**,**h**) Associations of TIMP1 tumor cell histoscore with (**e**) CSS and (**h**) OS. (**f**, **i**) Associations of TIMP1 other cell histoscore with (**f**) CSS and (**i**) OS. (**g**,**j**) Associations of TIMP1 serum concentrations with (**g**) CSS and (**j**) OS.

Finally, we analyzed the prognostic potential of tissue TIMP1 expression and serum TIMP1 levels. The results from ROC and Kaplan-Meier analyses are shown in Fig. 2. The AUC values of ROC curves varied between 0.535 and 0.591, indicating relatively weak discrimination among patients. Additional metrics for tissue and serum TIMP1 as prognostic biomarkers (such as true and false positive and negative rate and accuracy) are provided in Table S3.

Among patients with TIMP1 IHC data, there were 238 deaths (31.7%), including 133 cancer deaths, during the 10-year follow-up. ROC analyses showed that TIMP1 histoscore in stromal cells had higher AUC values than TIMP1 histoscore in tumor cells. Based on the ROC curves, cut-off values of 54.2 and 76.7 were chosen for stromal and tumor histoscores, respectively. Kaplan-Meier survival functions showed higher probabilities of CSS and OS for patients with higher TIMP1 histoscore in stromal cells (log rank *p* < 0.001 for both). In contrast, no differences in survival were observed between groups stratified by TIMP1 histoscore in tumor cells. Cox regression analyses (Table 3) showed a similar association between high TIMP1 histoscore in stromal cells and improved survival in univariable analysis (CSS: HR 0.53, 95% CI 0.37–0.75; OS: HR 0.69, 95% CI 0.53–0.90). However, these associations were not significant after adjustment for clinicopathological features in multivariable models (CSS: HR 0.91, 95% CI 0.62–1.35; OS: HR 0.99, 95% CI 0.74–1.34).

**Table 3:** Univariable and multivariable Cox regression models for cancer-specific survival and overall survival according to TIMP1 histoscore and TIMP1 serum levels.

	No. of cases	Colorectal cancer-specific survival			Overall survival		
		No. of events	Univariable HR (95% CI)	Multivariable HR (95% CI)	No. of events	Univariable HR (95% CI)	Multivariable HR (95% CI)
TIMP1 histoscore in tumor cells							
≤ 76.7	227	50	1 (referent)	1 (referent)	85	1 (referent)	1 (referent)
> 76.7	525	83	0.79 (0.56–1.13)	1.08 (0.74–1.57)	153	0.92 (0.70–1.20)	1.13 (0.85 − 0.15)
*P*			0.20	0.68		0.52	0.39
TIMP1 histoscore in stromal cells							
≤ 54.2	193	55	1 (referent)	1 (referent)	87	1 (referent)	1 (referent)
> 54.2	559	78	0.53 (0.37–0.75)	0.91 (0.62–1.35)	151	0.69 (0.53–0.90)	0.99 (0.74–1.34)
*P*			< 0.001	0.66		0.006	0.97
Serum TIMP1							
≤ 399.7 ng/ml	494	67	1 (referent)	1 (referent)	115	1 (referent)	1 (referent)
> 399.7 ng/ml	108	23	2.05 (1.27–3.30)	1.29 (0.77–2.16)	49	2.66 (1.90–3.73)	1.85 (1.30–2.65)
*P*			0.003	0.34		< 0.001	< 0.001

Multivariable Cox proportional hazards regression models were adjusted for sex, age (< 65, 65–75, > 75), year of operation (2006–2010, 2011–2015, 2016–2020), tumor location (proximal colon, distal colon, rectum), disease stage (I–II, III, IV), tumor grade (well/moderately differentiated, poorly differentiated), lymphovascular invasion (negative, positive), mismatch repair (MMR) status (proficient, deficient), *BRAF* status (wild-type, mutant). The missing data in the TIMP1 serum model (*n* = 6 for *BRAF* status) were included in the majority category (*BRAF* wild-type) to limit the degrees of freedom.

*CI* confidence interval, *HR* hazard ratio.

Among patients with TIMP1 serum data, there were 164 deaths (27.2%), including 90 cancer deaths, during the 10-year follow-up. ROC analysis suggested that TIMP1 serum levels had greater prognostic value for OS than CSS. A cut-off value of 339.7 µg/ml was chosen for survival analyses. Kaplan-Meier survival functions showed lower survival probabilities for patients with higher serum TIMP1 values (CSS log rank *p* = 0.003, OS log rank *p* < 0.001). Cox regression analyses (Table 3) showed an association between higher TIMP1 serum values and worse survival in univariable analysis (CSS: HR 2.05, 95% CI 1.27–3.30; OS: HR 2.66, 95% CI 1.90–3.73). In multivariable analysis adjusted for other established prognostic parameters such as disease stage, MMR status, and lymphovascular invasion, the association between high serum TIMP1 levels and worse OS remained statistically significant (HR 1.85, 95% CI 1.30–2.65).

Complete multivariable Cox regression models of CSS and OS according to tissue and serum TIMP1 alongside other covariates can be seen in Tables S4-S6.

## Discussion

In this study, we examined the significance of both TIMP1 serum levels and TIMP1 tissue expression patterns in CRC. We discovered that TIMP1 expression in cancer tissue was correlated with T cell infiltration. Stromal TIMP1 correlated positively with overall T cell densities, while TIMP1 expression in cancer cells showed inverse correlation with T cell densities in the tumor center—suggesting divergent associations of TIMP1 with T cell densities depending on the location of TIMP1 expression and T cells in the tumor microenvironment. TIMP1 serum levels were correlated with the markers of systemic inflammation, and high TIMP1 serum values independently associated with shorter OS. These findings highlight the multifaceted role of TIMP1 in colorectal cancer that may depend on the site of action (tumor epithelium, tumor stroma, circulation).

We hypothesized that higher TIMP1 expression in stromal cells might be associated with a better disease outcome due to the protease inhibitory functions of TIMP1^1^. Supporting this, we discovered that higher TIMP1 expression in the stroma associated with a decrease in lymphovascular invasion and better survival in univariable models. In the tumor microenvironment, immune cells contribute to TIMP1 secretion^3^. Therefore, the positive correlation of stromal TIMP1 expression with T cell densities in our study could be an indication of enhanced immune cell infiltration.

In contrast, TIMP1 expression in tumor cells showed a negative correlation T cell densities the tumor center, supporting the hypothesis that TIMP1 in carcinoma cells may act as a pro-tumor cytokine. Previous studies have highlighted the anti-apoptotic and growth-factor like features of TIMP1 and suggested that its expression in tumor cells may enable cancer to evade immune responses, and create an immunosuppressive microenvironment^4^. Our observation of an inverse correlation between tumor cell TIMP1 expression tumor necrosis percentage aligns with this view, as TIMP1 may protect hypoxic cancer cells from dying. However, increased TIMP1 expression in tumor cells was not associated with increased disease stage or lymphovascular invasion, suggesting that its influence may be more localized to microenvironmental interactions rather than overt tumor progression.

Systemic inflammation in cancer involves a complex, multifaceted processes that remain incompletely understood. TIMP1 can trigger monocyte and neutrophil activation and cytokine production^12,25^, and hence TIMP1 is a potential tumor-derived factor driving systemic inflammation in CRC. In this study, serum TIMP1 levels were elevated in systemic inflammation, as indicated by their correlations with markers such as NLR, CRP, and IL6. Necrotic regions within tumors showed intensive TIMP1 staining, and serum TIMP1 levels positively correlated with tumor necrosis percentage. Although this correlation was relatively weak, TIMP1 released from necrotic tumor regions may contribute to the activation of circulating immune cells during systemic inflammation. Instead, tumor stromal or epithelial TIMP1 expression were not associated with serum TIMP1 levels.

Oncogenic *BRAF* mutations, high-level MSI, and proximal location are features frequently associated with colorectal cancer originating from sessile serrated lesions^26^. Colorectal cancers developing via serrated pathway have variable prognosis. Microsatellite stable, *BRAF* -mutated cancers have poor prognosis in general, whereas MSI-H reverts this^26–28^. In this study, *BRAF* status and proximal tumor location associated with both high TIMP1 serum levels and TIMP1 expression in tumor tissue. An association between elevated TIMP1 concentrations and *BRAF* mutations has been found in thyroid cancer, but not in CRC^29,30^. Larger studies are required to assess whether the prognostic role of TIMP1 might differ according to *BRAF* mutation status.

As our main analysis, we evaluated the prognostic value of TIMP1 serum levels and tissue expression. Higher TIMP1 serum levels associated with increased mortality in our cohort, which is the largest—to our knowledge—evaluating the prognostic value of TIMP1 levels in CRC. These results align with previous studies^29^. Notably, while high serum TIMP1 levels were associated with older age, they independently predicted shorter OS in the multivariable model adjusted for age. Beyond age, high serum TIMP1 levels were strongly associated with higher ASA classification, reflecting age-related conditions that contribute to general health. High TIMP1 levels have been associated with many conditions such as heart diseases^31^ and inflammatory diseases^3^ that may contribute to mortality in CRC patients. However, we did not observe any association between serum TIMP1 levels and coronary artery disease, diabetes, asthma, or COPD among CRC patients. Nevertheless, the results indicate that TIMP1 could represent a useful serum marker to predict overall mortality of CRC patients.

In contrast to some previous studies that reported associations between elevated TIMP1 levels in tumor tissue and poor prognosis in CRC^8,13^, our results showed no association between tumor cell TIMP1 expression and survival. However, higher stromal TIMP1 histoscore was linked to longer survival in univariable survival analysis, though this association did not remain significant in multivariable Cox regression models, suggesting that it was outperformed by other known prognostic factors. The observed positive survival effect of stromal TIMP1 expression differs from prior studies^8,13^, which have not distinguished between TIMP1 expression in epithelial and stromal cells^8^. Our results suggest that stromal TIMP1 expression, specifically, may be associated with better outcome. We hypothesize that this could be attributed to the positive correlation of stromal TIMP1 with immune cell densities, or its role as MMP inhibitor.

Our study had limitations. First, TIMP1 concentrations and immune cell densities were defined using TMAs, which only represent small tumor areas. However, prior research has shown the utility of TMAs in assessing immune infiltrates^20^. Second, data on postoperative cancer treatments were not available. Therefore, the predictive value of TIMP1 serum levels and tissue expression requires further study. Third, our study could have benefited from a validation cohort, and larger studies are required to assess the significance of TIMP1 in more specific patient subgroups such as those with *BRAF*-mutated tumors. Fourth, the manual assessment of histoscores for testing the reliability of the automated image analysis algorithms was challenging, particularly for stromal regions with lower cell densities. The strengths of the study include a large study population that contains extensive clinicopathological, histological, and serum biomarker data. Our use of supervised machine-learning algorithms to estimate TIMP1 staining intensities enabled accurate and consistent analysis of a large cohort. Validation analyses showed that these algorithms reliably distinguished tumor epithelial and stromal regions and calculated histoscores.

In conclusion, our study examined the prognostic role of TIMP1 in CRC, as well as its associations with host responses. We found that high serum TIMP1 values independently predict worse overall survival of colorectal cancer patients, thus reinforcing the utility of serum TIMP1 as a prognostic marker. TIMP1 expression in tumor epithelial and stromal cells show divergent associations with T cell infiltrates in tumors and patient outcome, suggesting that TIMP1 may harbor compartment-dependent functions within the tumor microenvironment.

## Electronic supplementary material

Below is the link to the electronic supplementary material.

Supplementary Material 1

## Data Availability

Data generated and/or analyzed during this study are not publicly available. The sharing of data will require approval from relevant ethics committees and/or biobanks. Further information including the procedures to obtain and access data of Finnish Biobanks are described at https://finbb.fi/en/fingenious-service.
