# Supplementary material for: Associations of serum and tissue TIMP1 with host response and survival in colorectal cancer
Source: Sci Rep. 2025 Jan 9;15:1440. doi: 10.1038/s41598-025-85549-3 (PMC11717928; doi:10.1038/s41598-025-85549-3)
Supplement: Supplementary file 1 — Supplementary Material 1 [file 41598_2025_85549_MOESM1_ESM.pdf]

Associations of serum and tissue TIMP1 with host response and survival in colorectal cancer

Supplementary online material

Akseli Kehusmaa, Anne Tuomisto, Päivi Sirniö, Henna Karjalainen, Meeri Kastinen, Vilja V. Tapiainen, Ville K. Äijälä, Taina Tervahartiala, Timo Sorsa, Jukka Rintala, Sanna Meriläinen, Juha Saarnio, Tero Rautio, Markus J. Mäkinen, Juha P. Väyrynen

6 tables, 1 figure

Abbreviations used in the manuscript:
AJCC, The American Joint Committee on Cancer; ASA, American Association of Anesthesiologists; AUC, area under the curve; CI, confidence interval; CRC, colorectal cancer; CRP, C-reactive protein; CSS, cancer-specific survival; CT, central tumor; DAB, 3,3'-Diaminobenzide; FFPE; formalin-fixed paraffin-embedded; HR, hazard ratio; IHC, immunohistochemistry; IM, invasive margin; mGPS, modified Glascow prognostic score; MSI, microsatellite instability; TIMP1, tissue inhibitor of metalloproteinases 1; MMR, mismatch repair; MMP, matrix metalloproteinase; NLR, neutrophil-to-lymphocyte ratio; OS, overall survival; ROC, receiver operating characteristics; TMA, tissue microarray

Table S1. Associations of tissue and serum TIMP1 with common comorbidities.

Characteristic	TIMP1 histoscore in tumors					TIMP1 serum		
	Total N	TIMP1 tumor cells Median (IQR)	*P*	TIMP1 stromal cells Median (IQR)	*P*	Total N	TIMP1 serum ng/ml Median (IQR)	*P*
All cases	757 (100%)	111 (66-156)		81 (53-110)		606 (100%)	317 (268-376)	
Diabetes			0.97		0.94			0.18
No	585 (77%)	111 (67-156)		80 (53-112)		468 (77%)	315 (266-375)	
Yes	172 (23%)	107 (64-156)		82 (54-109)		138 (23%)	323 (278-386)	
Coronary artery disease			0.57		0.76			0.62
No	602 (80%)	110 (66-155)		80 (54-110)		487 (80%)	318 (270-376)	
Yes	155 (20%)	116 (67-156)		83 (51-113)		119 (20%)	311 (266-375)	
Asthma			0.67		0.74			0.88
No	656 (87%)	107 (67-155)		80 (53-112)		515 (85%)	317 (269-376)	
Yes	101 (13%)	117 (66-157)		84 (62-106)		91 (15%)	321 (268-377)	
COPD			0.058		0.31			0.32
No	728 (96%)	108 (66-155)		80 (53-111)		581 (96%)	317 (268-375)	
Yes	29 (4%)	137 (99-165)		90 (78-105)		25 (4%)	335 (271-405)	

Table S2. Correlations between TIMP1 histoscore in tumors (tumor cells and stromal cells), TIMP1 serum levels, histological features, and systemic inflammation markers.

Variable	TIMP1 tumor center									TIMP1 invasive margin								
	N	TIMP1 histoscore tumor cells CT				TIMP1 histoscore stromal cells CT				N	TIMP1 histoscore tumor cells IM				TIMP1 histoscore stromal cells IM			
		Unadjusted		Adjusted		Unadjusted		Adjusted			Unadjusted		Adjusted		Unadjusted		Adjusted	
		Pearson r	p value	Beta	p value	Pearson r	p value	Beta	p value		Pearson r	p value	Beta	p value	p value	p value	Beta	p value
CD3+ T cell density (CT, epithelial)	753	-0.062	0.091	-0.13	<0.001	0.016	0.66	-0.065	0.092	741	-0.070	0.056	-0.12	0.002	0.044	0.24	-0.019	0.63
CD3+ T cell density (CT, stromal)	753	-0.13	<0.001	-0.15	<0.001	-0.068	0.75	-0.097	0.010	741	-0.14	<0.001	-0.15	<0.001	0.003	0.93	-0.014	0.72
CD3+ T cell density, (IM, epithelial)	746	0.021	0.57	-0.017	0.66	0.12	<0.001	0.076	0.044	738	-0.016	0.66	-0.048	0.21	0.085	0.020	0.034	0.38
CD3+ T cell density (IM, stromal)	746	0.011	0.77	-0.014	0.72	0.11	0.002	0.076	0.045	738	-0.045	0.22	-0.063	0.10	0.11	0.002	0.081	0.037
CD8+ T cell density (CT, epithelial)	753	-0.034	0.35	-0.11	0.006	0.074	0.043	-0.015	0.70	741	-0.038	0.30	-0.095	0.016	0.090	0.014	0.022	0.58
CD8+ T cell density (CT, stromal)	753	-0.036	0.33	-0.094	0.013	0.081	0.026	0.009	0.82	741	-0.065	0.077	-0.11	0.003	0.087	0.018	0.029	0.44
CD8+ T cell density (IM, epithelial)	748	0.017	0.64	-0.043	0.27	0.098	0.008	0.019	0.62	739	-0.022	0.55	-0.079	0.044	0.10	0.010	0.023	0.55
CD8+ T cell density (IM, stromal)	748	0.049	0.18	0.18	0.86	0.17	<0.001	0.011	0.003	739	-0.012	0.76	-0.054	0.16	0.15	<0.001	0.098	0.010

Abbreviations: CT, tumor center; IM, invasive margin. The adjusted beta coefficients and P values were calculated with linear regression models that included age (<65, ≥65), sex (male, female), tumor localization (colon, rectum), T class (T1-T2, T3-T4), N class (N0, N1-N2), M class (M0, M1), *BRAF* status (wild-type, mutant), MMR status (proficient, deficient), and tumor grade (low, high). Continuous variables that were not normally distributed were logarithmically transformed.

Table S3. Prognostic parameters for tissue and serum TIMP1 as biomarkers.

	Colorectal cancer-specific survival			Overall survival		
Parameter	TIMP1 histoscore in tumor cells (cut-off 76.7)	TIMP1 histoscore in stromal cells (cut-off 54.2)	Serum TIMP1 (cut-off 399.7 ng/ml)	TIMP1 histoscore in tumor cells (cut-off 76.7)	TIMP1 histoscore in stromal cells (cut-off 54.2)	Serum TIMP1 (cut-off 399.7 ng/ml)
True positive rate	0.38	0.41	0.26	0.36	0.34	0.30
True negative rate	0.71	0.78	0.83	0.72	0.79	0.86
False positive rate	0.62	0.59	0.74	0.64	0.66	0.70
False negative rate	0.29	0.22	0.17	0.28	0.21	0.14
Accuracy	0.65	0.71	0.75	0.61	0.66	0.71

Table S4 Multivariable Cox regression models of cancer-specific survival and overall survival according to TIMP1 serum levels and other covariates.

	Cancer-specific survival	Overall survival
	Multivariable HR (95% CI)	Multivariable HR (95% CI)
TIMP1 serum concentration		
≤399.7 ng/ml	1 (referent)	1 (referent)
>399.7 ng/ml	1.29 (0.77-2.16)	1.85 (1.30-2.65)
Age		
<65	1 (referent)	1 (referent)
65-75	1.17 (0.68-2.03)	1.33 (0.84-2.11)
>75	2.44 (1.40-4.26)	3.86 (2.50-5.96)
Gender		
Male	1 (referent)	1 (referent)
Female	0.74 (0.47-1.17)	0.75 (0.54-1.03)
Year of operation		
2006-2010	1 (referent)	1 (referent)
2011-2015	1.47 (0.52-4.14)	0.96 (0.52-1.77)
2016-2020	0.7 (0.24-2.04)	0.60 (0.32-1.15)
Tumor location		
Proximal colon	1 (referent)	1 (referent)
Distal colon	1.13 (0.65-1.95)	1.21 (0.79-1.85)
Rectum	0.92 (0.53-1.58)	1.08 (0.72-1.64)
AJCC disease stage		
I-II	1 (referent)	1 (referent)
III	2.92 (1.51-5.67)	1.21 (0.81-1.80)
IV	24.03 (11.65-49.54)	7.73 (4.72-12.66)
Tumor grade		
Low-grade	1 (referent)	1 (referent)
High-grade	1.70 (0.99-2.93)	1.38 (0.90-2.10)
Lymphovascular invasion		
No	1 (referent)	1 (referent)
Yes	1.51 (0.86-2.65)	1.39 (0.95-2.04)
MMR status		
MMR proficient	1 (referent)	1 (referent)
MMR deficient	0.48 (0.17-1.35)	0.91 (0.51-1.63)
BRAF status		
Wild-type	1 (referent)	1 (referent)
Mutant	1.02 (0.46-2.26)	1.19 (0.69-2.04)

Abbreviations: AJCC, American Joint Committee on Cancer; CI, confidence interval; HR, hazard ratio; MMR, mismatch repair. Missing data for *BRAF* status (*n*=6) values were replaced with the most frequent value (wild-type) to limit the degrees of freedom.

Table S5 Multivariable Cox regression models of cancer-specific survival and overall survival according to TIMP1 histoscore in tumor cells and other covariates.

	Cancer-specific survival	Overall survival
	Multivariable HR (95% CI)	Multivariable HR (95% CI)
TIMP1 histoscore in tumor cells		
≤76.7	1 (referent)	1 (referent)
>76.7	1.08 (0.74-1.57)	1.13 (0.85-1.50)
Age		
<65	1 (referent)	1 (referent)
65-75	1.49 (0.95-2.32)	1.55 (1.06-2.26)
>75	2.43 (1.56-3.81)	4.11 (2.89-5.85)
Gender		
Male	1 (referent)	1 (referent)
Female	0.92 (0.65-1.32)	0.79 (0.61-1.03)
Year of operation		
2006-2010	1 (referent)	1 (referent)
2011-2015	0.83 (0.55-1.24)	0.80 (0.59-1.09)
2016-2020	0.38 (0.23-0.61)	0.49 (0.34-0.69)
Tumor location		
Proximal colon	1 (referent)	1 (referent)
Distal colon	1.19 (0.76-1.86)	1.15 (0.81-1.62)
Rectum	0.94 (0.60-1.48)	1.09 (0.77-1.52)
AJCC disease stage		
I-II	1 (referent)	1 (referent)
III	3.12 (1.79-5.44)	1.30 (0.93-1.82)
IV	22.19 (12.40-39.71)	8.06 (5.48-11.84)
Tumor grade		
Low-grade	1 (referent)	1 (referent)
High-grade	1.67 (1.07-2.60)	1.25 (0.87-1.80)
Lymphovascular invasion		
No	1 (referent)	1 (referent)
Yes	1.78 (1.11-2.83)	1.40 (1.03-1.91)
MMR status		
MMR proficient	1 (referent)	1 (referent)
MMR deficient	0.52 (0.24-1.13)	0.97 (0.61-1.54)
BRAF status		
Wild-type	1 (referent)	1 (referent)
Mutant	1.52 (0.81-2.84)	1.34 (0.85-2.11)

Abbreviations: AJCC, American Joint Committee on Cancer; CI, confidence interval; HR, hazard ratio; MMR, mismatch repair.

Table S6 Multivariable Cox regression models of cancer-specific survival and overall survival according to TIMP1 histoscore in stromal cells and other covariates.

	Cancer-specific survival	Overall survival
	Multivariable HR (95% CI)	Multivariable HR (95% CI)
TIMP1 histoscore in stromal cells		
≤54.2	1 (referent)	1 (referent)
>54.2	0.91 (0.62-1.35)	0.99 (0.74-1.34)
Age		
<65	1 (referent)	1 (referent)
65-75	1.50 (0.96-2.34)	1.56 (1.07-2.28)
>75	2.44 (1.56-3.83)	4.11 (2.89-5.86)
Gender		
Male	1 (referent)	1 (referent)
Female	0.91 (0.64-1.30)	0.78 (0.60-1.02)
Year of operation		
2006-2010	1 (referent)	1 (referent)
2011-2015	0.84 (0.56-1.26)	0.81 (0.60-1.11)
2016-2020	0.40 (0.24-0.65)	0.51 (0.35-0.73)
Tumor location		
Proximal colon	1 (referent)	1 (referent)
Distal colon	1.18 (0.75-1.84)	1.13 (0.80-1.60)
Rectum	0.92 (0.58-1.46)	1.08 (0.77-1.52)
AJCC disease stage		
I-II	1 (referent)	1 (referent)
III	3.13 (1.80-5.47)	1.31 (0.94-1.84)
IV	22.07 (12.31-39.55)	8.08 (5.49-11.89)
Tumor grade		
Low-grade	1 (referent)	1 (referent)
High-grade	1.72 (1.09-2.70)	1.26 (0.87-1.81)
Lymphovascular invasion		
No	1 (referent)	1 (referent)
Yes	1.75 (1.09-2.80)	1.39 (1.02-1.90)
MMR status		
MMR proficient	1 (referent)	1 (referent)
MMR deficient	0.52 (0.24-1.13)	0.97 (0.61-1.55)
BRAF status		
Wild-type	1 (referent)	1 (referent)
Mutant	1.56 (0.84-2.91)	1.37 (0.87-2.16)

Abbreviations: AJCC, American Joint Committee on Cancer; CI, confidence interval; HR, hazard ratio; MMR, mismatch repair.

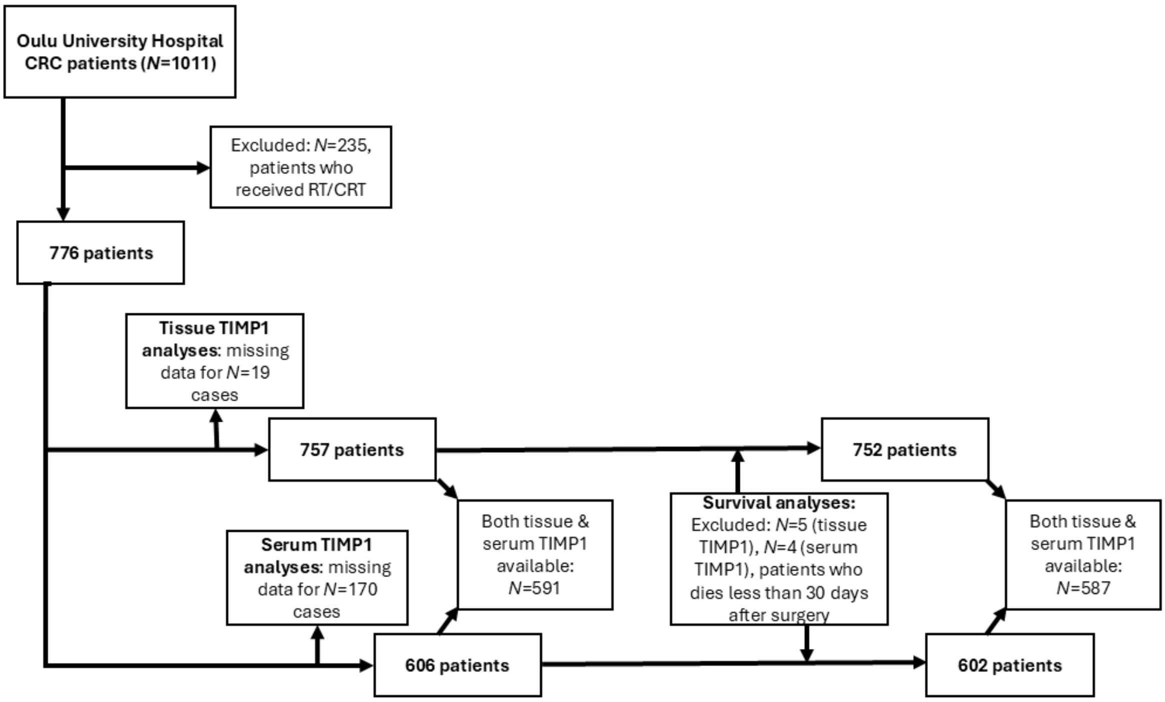

Fig. S1. Flowchart of the patients analyzed in the study. Abbreviations: RC, radiotherapy; CRT chemoradiotherapy.
